# Supplementary material for: Peak refreezing in the Greenland firn layer under future warming scenarios
Source: Nat Commun. 2022 Nov 11;13:6870. doi: 10.1038/s41467-022-34524-x (PMC9652464; doi:10.1038/s41467-022-34524-x)
Supplement: Supplementary file 1 — Supplementary Information [file 41467_2022_34524_MOESM1_ESM.pdf]

Supplementary Information: "Peak refreezing in the Greenland firn layer under future warming scenarios"

Brice Noël*[1], Jan T. M. Lenaerts[2], William H. Lipscomb[3], Katherine Thayer-Calder[3], and Michiel R. van den Broeke[1]

[1]*Institute for Marine and Atmospheric research Utrecht, Utrecht University, Utrecht, Netherlands.*
[2]*Department of Atmospheric and Oceanic Sciences, University of Colorado Boulder, Boulder, CO, USA.*
[3]*Climate and Global Dynamics Laboratory, National Center for Atmospheric Research, Boulder, CO, USA.*

This document includes:

- Supplementary Figures 1-9
- Supplementary Tables 1-2
- Supplementary References

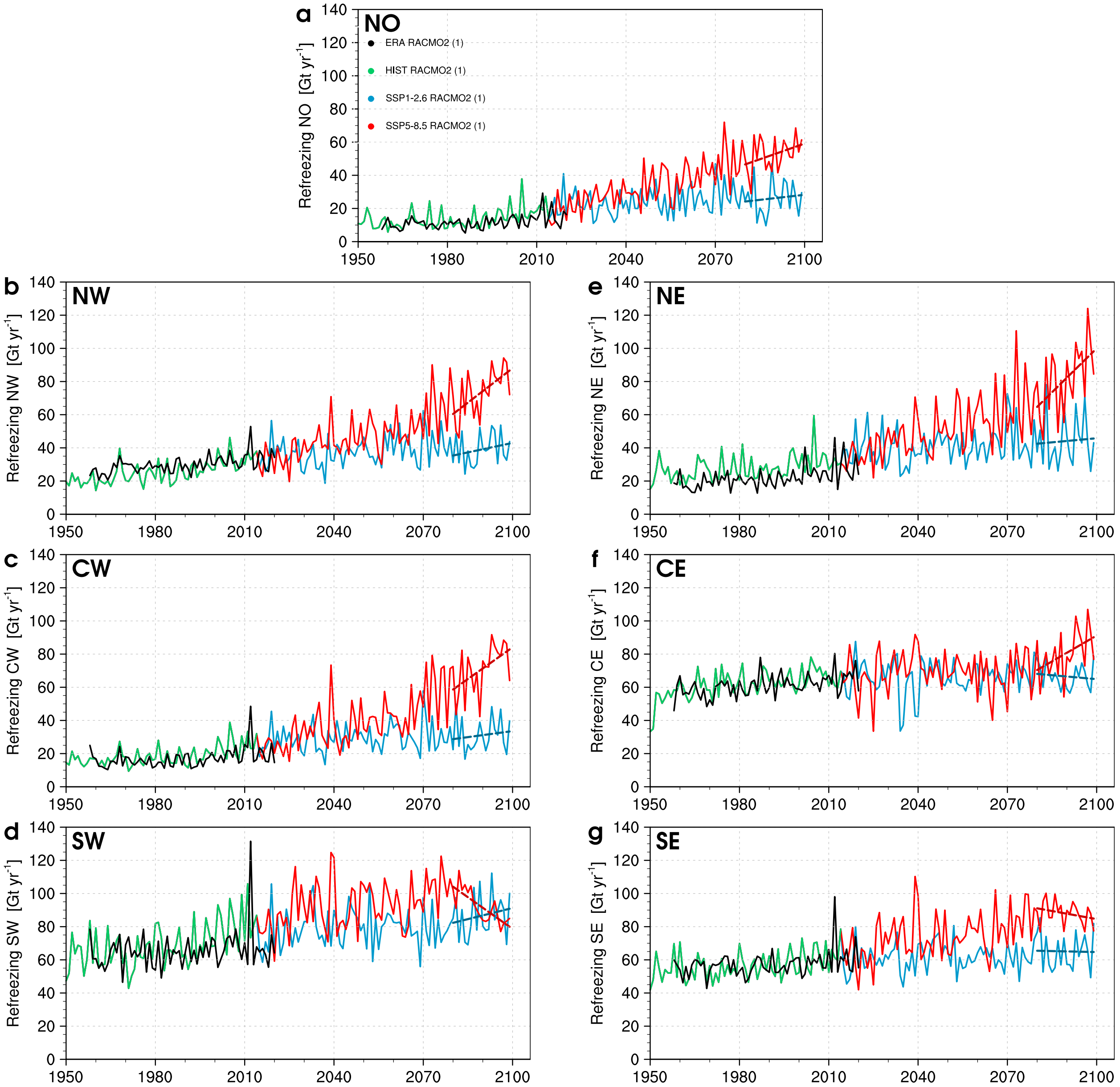

Supplementary Figure 1: Evolution of firn refreezing by sector until 2100. **a-g** Time series of refreezing spatially integrated over the individual sectors of Greenland for the historical, SSP1-2.6 and SSP5-8.5 CESM2-forced RACMO2.3p2 simulations. The black line in **a-g** represents the reanalysis-forced RACMO2.3p2 simulation (1958-2020)[1].

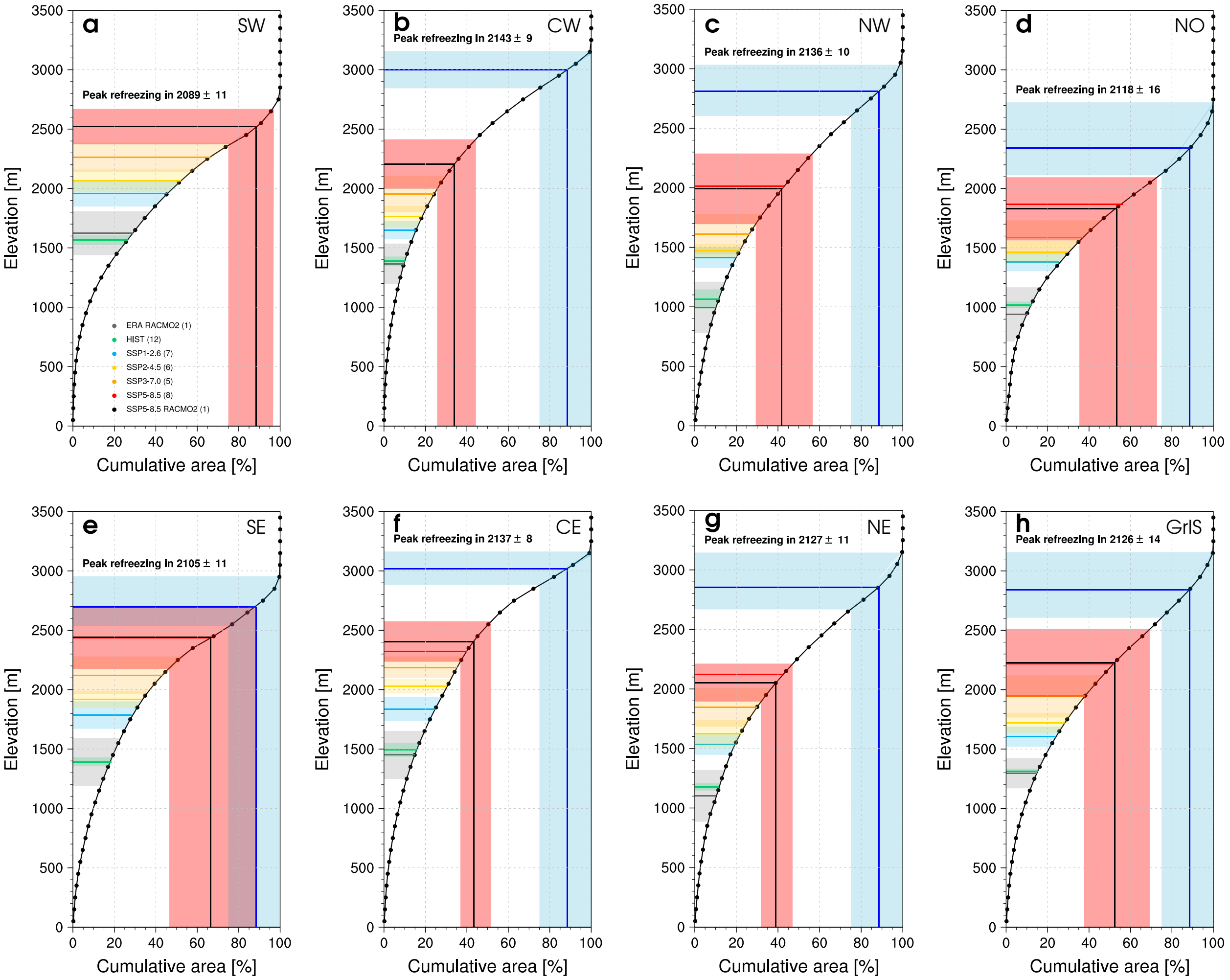

Supplementary Figure 2: Regional hypsometry and threshold runoff line altitude. **a-h** Hypsometry of individual sectors of the GrIS, i.e., the fraction of glacier area per 100 m elevation bins. Each panel shows the (regional) ensemble-mean runoff line altitude from 12 historical simulations (1960-1990) and 27 projections under SSP1-2.6 (6 CESM2 and 1 RACMO2 members), SSP2-4.5 (6 members), SSP3-7.0 (5 members) and SSP5-8.5 (8 CESM2 and 1 RACMO2 members), and from the CESM2-forced RACMO2.3p2 under SSP5-8.5 (black) for the period 2080-2099. The grey line represents the mean runoff line altitude from the reanalysis-forced RACMO2.3p2 simulation for 1960-1990[1]. The blue line shows the threshold runoff line altitude marking the (regional) peak refreezing when $90 \pm 10\%$ of a sector area produces significant runoff ($>$100 mm w.e. yr^{-1}). Coloured bands represent the uncertainty estimated as two standard deviations of the runoff line altitude for the corresponding period. Each panel estimates the crossing of the (regional) threshold runoff line altitude under SSP5-8.5.

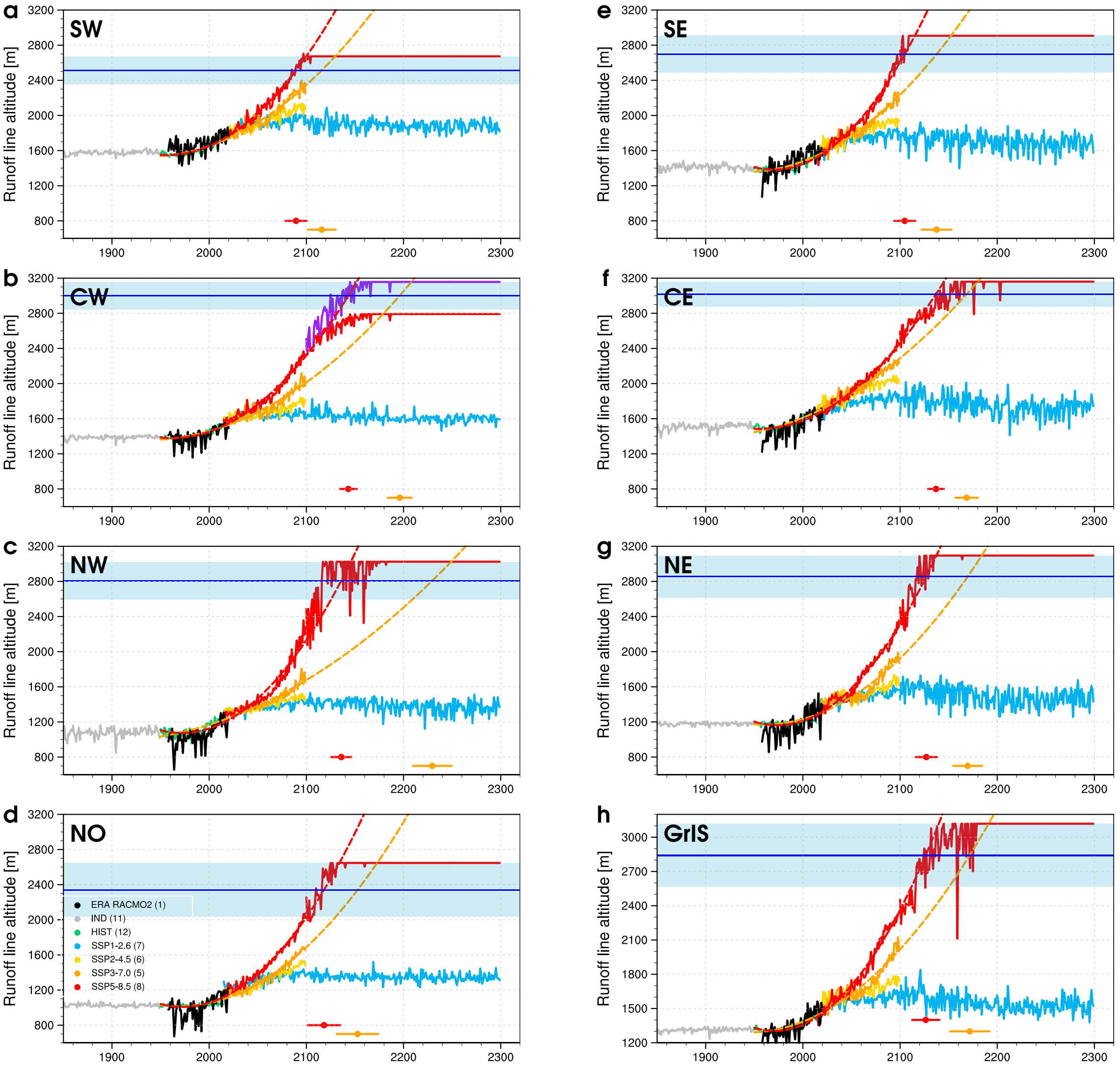

Supplementary Figure 3: Predicted crossing of a regional threshold runoff line altitude. **a-h** Time series of regional and GrIS ensemble-mean runoff line altitude from 11 pre-industrial (IND), 12 historical (HIST) and 27 scenario projections, i.e., SSP1-2.6 (6 CESM2 and 1 RACMO2 members), SSP2-4.5 (6 members), SSP3-7.0 (5 members), and SSP5-8.5 (7 CESM2 and 1 RACMO2 members). Note that the CESM2 SSP5-8.5 scenario including ice dynamics is not shown. The blue line shows the threshold runoff line altitude marking the (regional) peak refreezing, and the blue band represents the associated uncertainty. Dashed coloured lines are quadratic regressions based on the period 1950-2099 for SSP5-8.5 (red) and SSP3-7.0 (orange) scenarios. Coloured dots and whiskers estimate the timing and uncertainties of the regional peak refreezing for SSP5-8.5 (red) and SSP3-7.0 (orange). Note that in CW Greenland, regression parameters from neighbouring CE sector (purple line; see Supplementary Fig. 3f) were used after 2100 instead of the CW ones (red line; see Supplementary Fig. 3b). The reason is that CW regression parameters, estimated for the period 1950-2099, artificially decrease the sector maximum elevation.

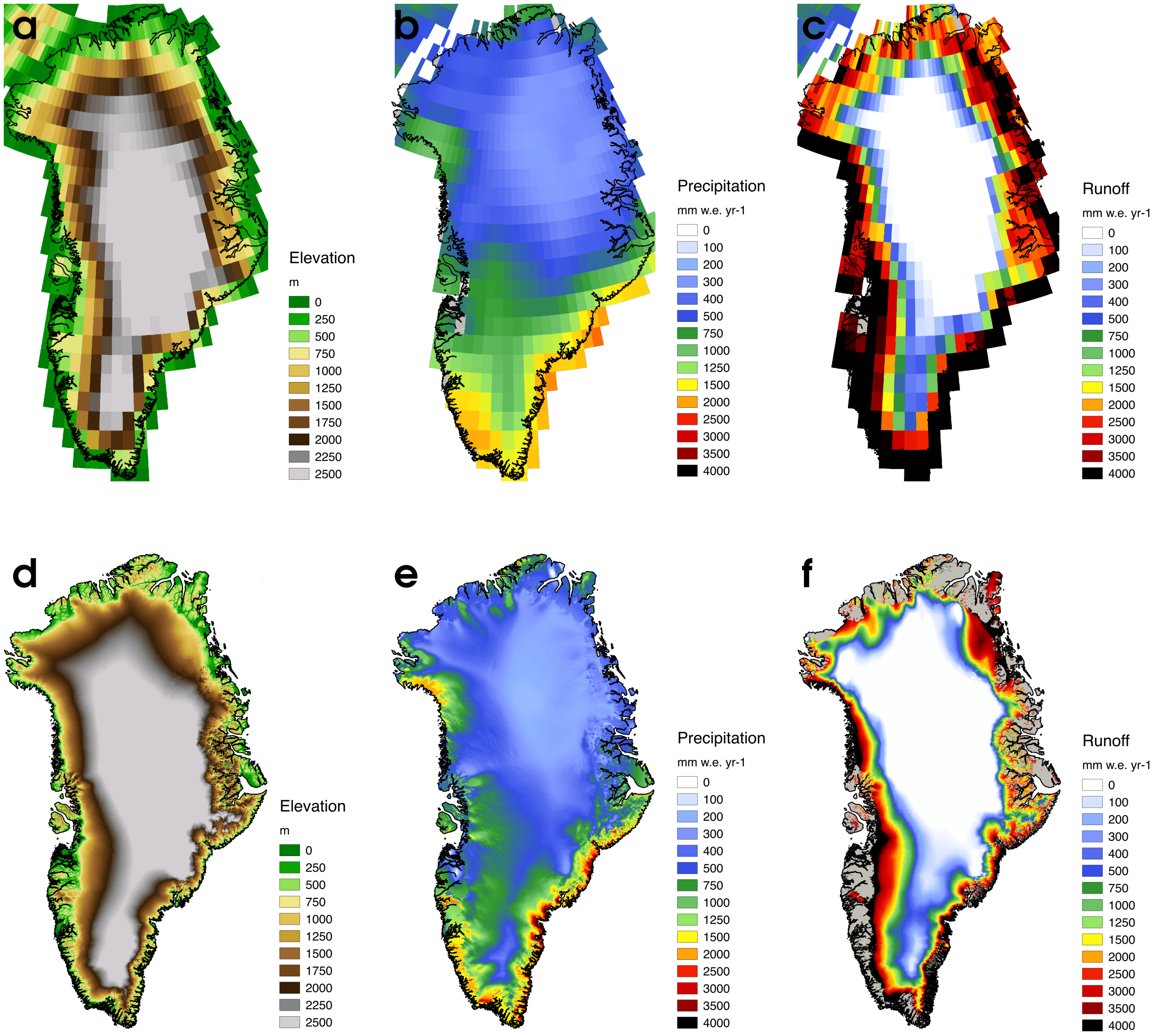

Supplementary Figure 4: Statistical downscaling of native CESM2 to 1 km resolution. **a** Topography of Greenland prescribed in native CESM2, **b** total precipitation and **c** meltwater runoff under SSP5-8.5 in 2099 at 0.9 x 1.25^o spatial resolution ($\sim$ 111 km). **d** Topography derived from the GIMP DEM[2] down-sampled to 1 km resolution. **e-f** same as **b-c** but statistically downscaled from the native CESM2 resolution to the 1 km grid.

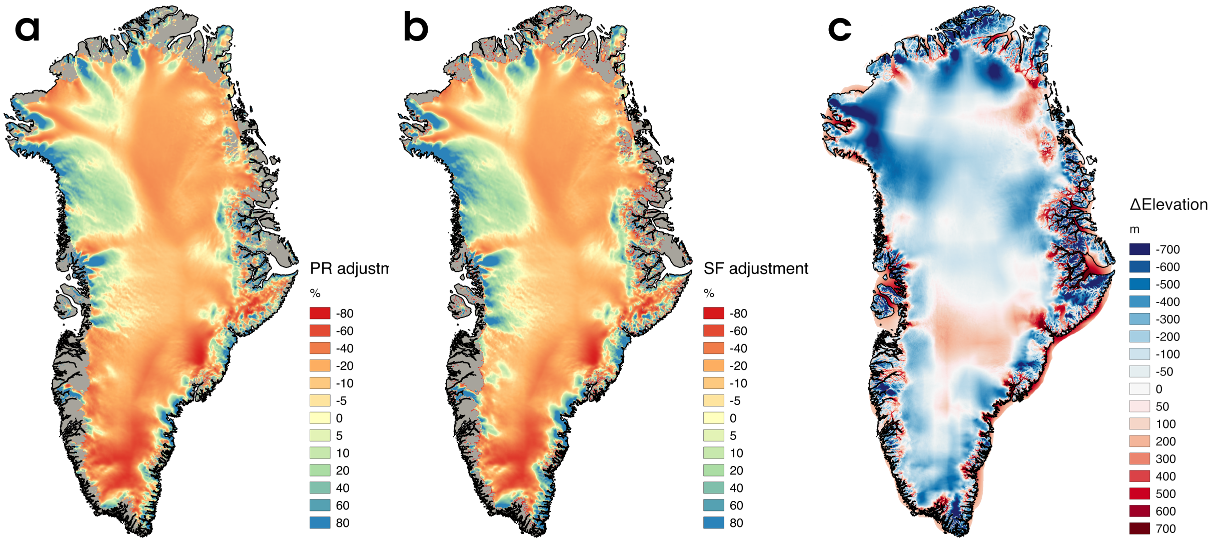

Supplementary Figure 5: Precipitation adjustment in CESM2. Adjustment factor for **a** total precipitation and **b** snowfall used to correct the spatial distribution of the native CESM2 precipitation fields. Adjustment factors are estimated using reanalysis-forced RACMO2 data at 5.5 km resolution (1958-2020)[1]. **c** Elevation difference between the $\sim$111 km topography prescribed in CESM2 and the GIMP DEM[2] down-sampled to 1 km.

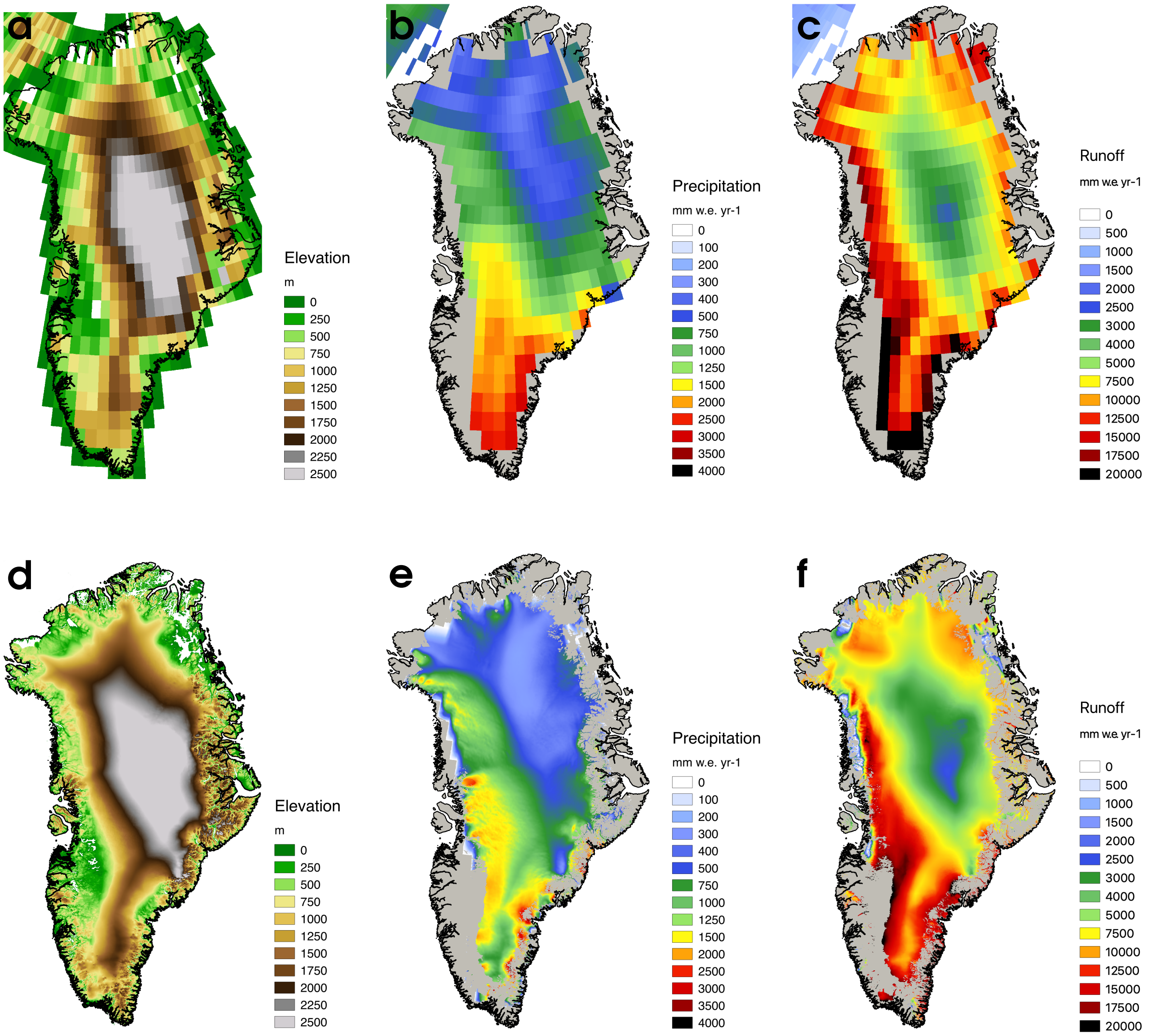

Supplementary Figure 6: Statistical downscaling of CESM2-CISM2 to 1 km resolution. **a** Topography of Greenland modelled by CESM2-CISM2 in 2299, **b** total precipitation and **c** meltwater runoff under SSP5-8.5 in 2299 at 0.9 x 1.25^o spatial resolution ($\sim$ 111 km). **d** Topography derived from the GIMP DEM[2], BedMachine v3[3] and CISM2 ice dynamics at 4 km, down-sampled to 1 km resolution. **e-f** same as **b-c** but statistically downscaled from the native CESM2-CISM2 resolution to the 1 km grid. Note the rapid ice sheet thinning and retreat, combined with enhanced runoff relative to the 2099 conditions (Supplementary Figure 4).

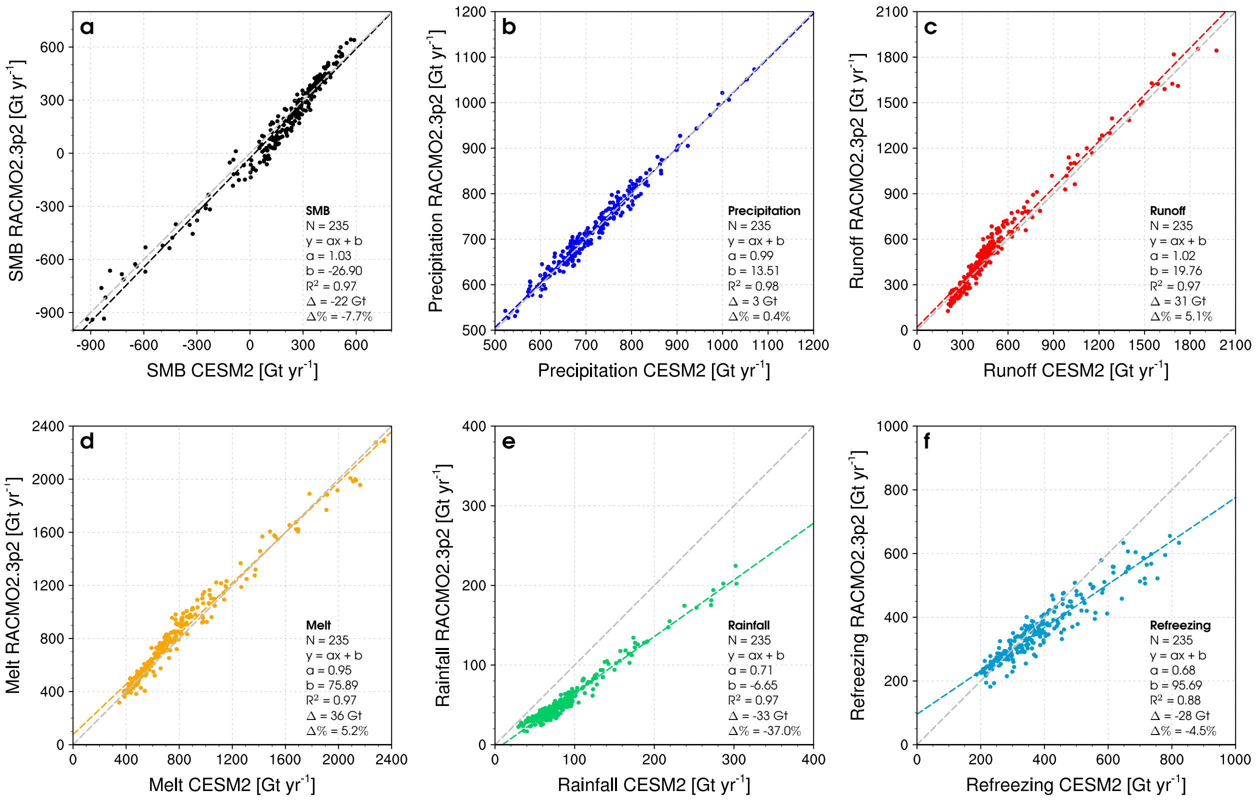

Supplementary Figure 7: Cross-model correlation of GrIS SMB and components. Correlation of GrIS-integrated **a** SMB, **b** total precipitation, **c** runoff, **d** melt, **e** rainfall and **f** refreezing between the CESM2-forced RACMO2.3p2 and the corresponding CESM2 products statistically downscaled to 1 km spatial resolution for the historical period (1950-2014) and for both SSP1-2.6 and SSP5-8.5 warming scenarios (2015-2099). Listed are number of years (N), slope (a) and intercept (b) of the linear regression, coordination coefficient (R^2), and model difference (RACMO2.3p2 minus CESM2) expressed as an absolute (Δ) and relative ($\Delta\%$) value.

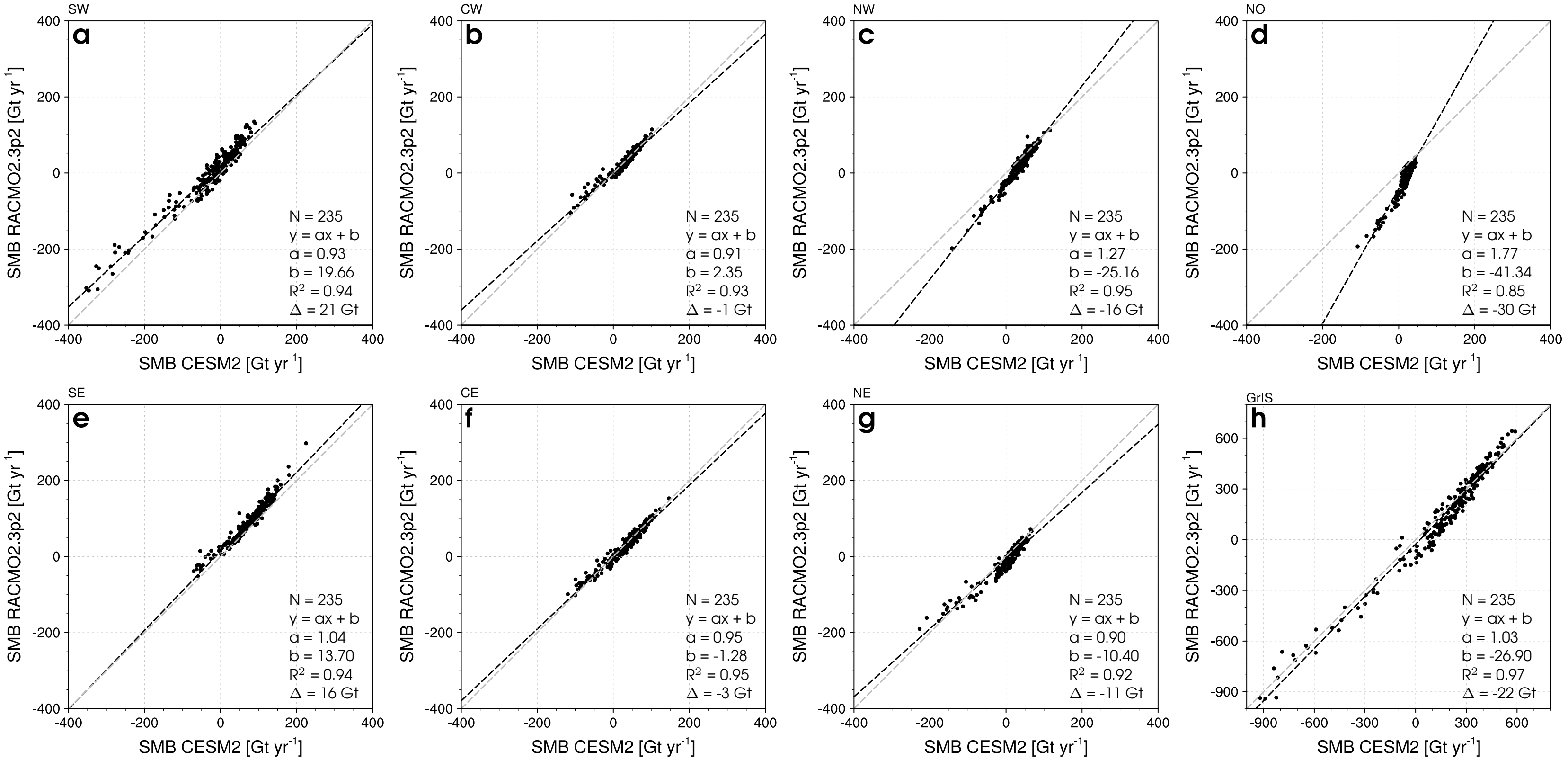

Supplementary Figure 8: Cross-model correlation of regional SMB. **a-h** Correlation of sector integrated SMB between the CESM2-forced RACMO2.3p2 and the corresponding CESM2 products statistically downscaled to 1 km spatial resolution for the historical period (1950-2014) and for both SSP1-2.6 and SSP5-8.5 warming scenarios (2015-2099). Listed are number of years (N), slope (a) and intercept (b) of the linear regression, coordination coefficient (R^2), and model difference (RACMO2.3p2 minus CESM2) expressed as an absolute value (Δ).

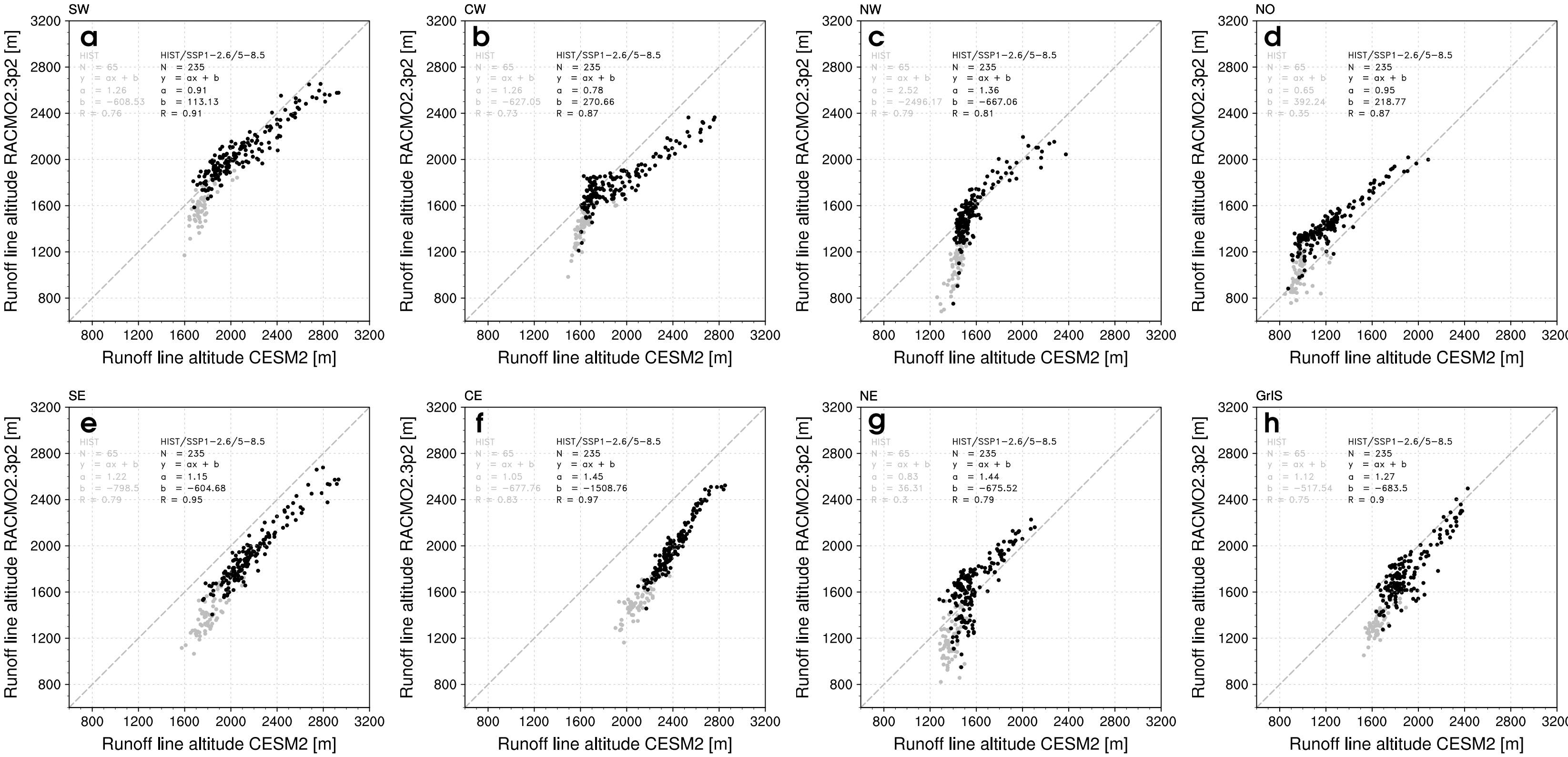

Supplementary Figure 9: Cross-model correlation of the runoff line altitude. **a-h** Correlation of (regional) runoff line altitude between the CESM2-forced RACMO2.3p2 and corresponding CESM2 product statistically downscaled to 1 km spatial resolution for the historical period (1950-2014; grey dots) and for both SSP1-2.6 and SSP5-8.5 warming scenarios (2015-2099; black dots). Number of years (N), slope (a) and intercept (b) of the linear regression, and correlation (R) are also listed for the historical period (grey) and the full data set (black).

Supplementary Table 1: Runoff line altitude and timing of the peak refreezing. Reference runoff line altitude in the period 1960-1989 for the historical simulations: HIST (12 CESM2 and 1 RACMO2 members). Runoff line altitude in the period 2080-2099 for different warming scenarios: SSP1-2.6 (6 CESM2 and 1 RACMO2 members), SSP2-4.5 (6 members), SSP3-7.0 (5 members) and SSP5-8.5 (8 CESM2 and 1 RACMO2 members). Threshold runoff line altitude marking a peak refreezing when 90 $\pm$ 10% of the sector area produces significant runoff (>100 mm w.e. yr^{-1}). Timing of the (regional) peak refreezing under SSP3-7.0 and SSP5-8.5 warming scenarios. Uncertainty quantification is explained in the Methods.

Runoff line	Units	NO	NW	NE	CW	CE	SW	SE	GrIS
Reference altitude	m a.s.l.	954 ± 209	1022 ± 293	1105 ± 232	1350 ± 238	1474 ± 173	1537 ± 228	1337 ± 181	1273 ± 129
SSP1-2.6	m a.s.l.	1431 ± 64	1496 ± 67	1591 ± 68	1713 ± 61	1838 ± 100	2007 ± 86	1821 ± 99	1649 ± 71
SSP2-4.5	m a.s.l.	1498 ± 77	1542 ± 42	1659 ± 88	1805 ± 72	2027 ± 58	2096 ± 77	1935 ± 61	1745 ± 69
SSP3-7.0	m a.s.l.	1600 ± 117	1648 ± 130	1827 ± 124	1956 ± 121	2184 ± 90	2253 ± 100	2107 ± 134	1934 ± 150
SSP5-8.5	m a.s.l.	1830 ± 190	1956 ± 263	2035 ± 175	2156 ± 154	2316 ± 169	2461 ± 168	2377 ± 254	2163 ± 118
Threshold altitude	m a.s.l.	2340 ± 307	2809 ± 215	2855 ± 239	3000 ± 157	3019 ± 141	2514 ± 159	2697 ± 210	2842 ± 276
Peak refreezing	Units	NO	NW	NE	CW	CE	SW	SE	GrIS
SSP3-7.0	year	2153 ± 21	2230 ± 20	2170 ± 15	2196 ± 12	2169 ± 12	2116 ± 14	2138 ± 15	2172 ± 20
SSP5-8.5	year	2118 ± 17	2136 ± 10	2127 ± 11	2143 ± 9	2137 ± 8	2089 ± 11	2105 ± 11	2126 ± 14

Supplementary Table 2: GrIS SMB and contribution to sea level rise by 2100. Annual mean GrIS-integrated runoff and SMB over the period 2080-2099 for different warming scenarios: SSP1-2.6 (6 CESM2 and 1 RACMO2 members), SSP2-4.5 (6 members), SSP3-7.0 (5 members) and SSP5-8.5 (8 CESM2 and 1 RACMO2 members). Uncertainty quantification is explained in the Methods. Negative SMB and uncertainties are converted into annual sea level rise equivalent (SLR), assuming that 362 Gt of ice raise global sea level by 1 mm.

2080-2099	Units	SSP1-2.6	SSP2-4.5	SSP3-7.0	SSP5-8.5
Runoff	Gt yr^{-1}	478 ± 38	596 ± 45	804 ± 104	1406 ± 268
SMB	Gt yr^{-1}	141 ± 45	76 ± 51	-101 ± 97	-594 ± 246
SLR	mm yr^{-1}	-	-	0.3 ± 0.3	1.6 ± 0.8

Supplementary References

1. Noël, B., van de Berg, W. J., Lhermitte, S. & van den Broeke, M. R. Rapid ablation zone expansion amplifies north Greenland mass loss. *Science Advances* **5**, eaaw0123 (2019).

2. Howat, I. M., Negrete, A. & Smith, B. E. The Greenland Ice Mapping Project (GIMP) land classification and surface elevation data sets. *The Cryosphere* **8**, 1509 – 1518 (2014).

3. Morlighem, M. *et al.* BedMachine v3: Complete Bed Topography and Ocean Bathymetry Mapping of Greenland From Multibeam Echo Sounding Combined With Mass Conservation . *Geophysical Research Letters* **44**, 11,051–11,061 (2017).
